# COVID-19 vaccination coverage among patients with psychiatric disorders in China during the pandemic: a cross-sectional study

**DOI:** 10.1186/s12888-022-04271-w

**Published:** 2022-10-26

**Authors:** Yue Qin, Ziru Zhao, Ziwei Teng, Baoyan Xu, Xianghe Wang, Jingyi Guo, Jing Huang, Haishan Wu

**Affiliations:** Department of Psychiatry, National Clinical Research Center for Mental Disorders, The Second Xiangya Hospital of Central South University, 410011 Changsha, Hunan China

**Keywords:** COVID-19, Vaccine acceptance, Vaccination rates, Psychiatric disorder, Schizophrenia

## Abstract

**Background:**

To investigate the Coronavirus Disease 2019 (COVID-19) vaccination coverage and the influential factors of vaccination among patients with mental disorders, we conducted a cross-sectional study in China.

**Method:**

The anonymous questionnaires including demographic data, vaccination status, intention to be vaccinated and its reasons were collected in the Second Xiangya Hospital, one of the biggest four psychiatric centers in China. Mental health of these participants were measured by the Patient Health Questionnaire (PHQ-9) and the Generalized Anxiety Disorder-7 items (GAD-7). The influential factors associated with vaccination status were analyzed by Fisher exact tests and binary logistical analysis.

**Result:**

1328 patients and 922 family members completed the survey. The vaccination rate of patients included was 69.4%, whereas 85.5% patients were willing to be vaccinated. Being hospitalized (aOR 0.41, 95% CI:0.27–0.60), suffering from schizophrenia (aOR 0.38, 95% CI: 0.19–0.75) and secondary school educational background (aOR 0.58, 95% CI: 0.37–0.93) were significantly associated with less likelihood to get vaccinated. Uptaking vaccines could reduce depressive (aOR 0.63, 95% CI: 0.41–0.98) or anxious symptoms (aOR 0.40, 95% CI: 0.25–0.63) in these patients for a short period.

**Conclusion:**

Further COVID-19 immunization programme should prioritize hospitalized psychiatric patients and schizophrenic patients since their demands for vaccination had been partly ignored during the current inoculation.

**Supplementary information:**

The online version contains supplementary material available at 10.1186/s12888-022-04271-w.

## Introduction

### Background

Coronavirus disease 2019 (COVID-19), caused by severe acute respiratory syndrome coronavirus 2 (SARS-CoV-2), continues to spread with its variant strains. By August 13, 2021, there had been 205 338 159 confirmed cases of COVID-19, including 4 333 094 deaths, as reported by the World Health Organization (WHO) [1]. The National Health Commission of China (NHCC) declared that the number of existing confirmed cases in the Chinese mainland was 1970 with a daily increase of 66 cases [2]. To control the epidemic, a series of precautionary measures, such as social distancing, wearing masks, governmental restrictions on public gathering and traveling, etc., have been deployed [3]. Additionally, to reduce virus transmission and mortality, COVID-19 vaccination of different types, including mRNA vaccines, vector vaccines, and inactivated vaccines, are being carried out around the world [4]. In China, people aged 12 and above were recommended to get vaccinated unless they were pregnant, allergic to the active ingredients of the vaccine, with uncontrolled epilepsy and other serious neurological diseases and suffering from an acute disease, acute exacerbation of chronic disease, or uncontrolled severe chronic disease [5]. As of August 12, 2021, 1 820 238 000 vaccine doses were administered in China and a total of 4 428 168 759 around the globe [6]. It had been reported that vaccines are effective in preventing COVID-19, including severe cases and COVID-19 related death [7]. Therefore, further promotion of vaccination and expansion of the population permitted to be vaccinated are urgently needed.

Previous studies have shown that SARS-CoV-2 infection increases the risk of anxiety and stress-related disorders, such as post-traumatic stress disorder (PTSD), leading to exacerbation of other psychiatric illnesses, including depression, bipolar disorder, schizophrenia, obsessive-compulsive disorder (OCD), substance abuse, and long-term cognitive decline [8–14]. Current studies have revealed high prevalence of depressive, anxiety, acute stress symptoms and increased COVID-19 related self-harm or suicidality in individuals with mental illness as well as an increased burden of nursing among their family members after the COVID-19 outbreak [15–18]. Moreover, patients in psychiatric institutes are more vulnerable to severe viral outbreaks because of their unclear movement trajectories before admission and low compliance to epidemic prevention measures like wearing masks after admission, easily leading to cluster infections in the wards [19]. Other outpatient psychiatric patients are also more likely to develop an infection because they are relatively incapable of practicing protective measures due to their disordered mental state, poor self-control and self-care, and inadequate insight, generated from an unhealthy lifestyle and the side effects of psychotropic medications [20]. The current study also showed that the death rate of COVID-19 patients with diagnosed mental illness is approximately 3.8% higher than those without psychoses. As a susceptible population, vaccination of patients with mental illness should be considered [21]. Several studies have revealed a range of side effects of vaccines, including fatigue, headache, tiredness, muscle pain, chills, fever, and nausea, but they are nearly always mild and transient, and can be viewed positively as a necessary prelude to an effective immune response [22]. Besides, except for insomnia that had been revealed affecting 1% vaccinated people, other symptoms related to mental health were not reported as adverse effects of COVID-19 vaccines [23]. However, a reported case has shown that COVID-19 vaccines might elevate clozapine levels and toxic effects, corresponding to worries about psychotropic medication in patients with mental illness [24]. Therefore, it is necessary to investigate whether vaccination could influence the psychological status of patients with mental disorders.

Current studies on vaccination status are primary among populations such as children and the elderly, but are rarely done in psychiatric patients. Studies conducted before vaccines were licensed showed that people with mental disorders displayed great likelihood to accept vaccination or pay for vaccines, which is associated with high vaccine coverage, resurgence of the pandemic, belief in the effectiveness of vaccines, and their personal risk profile [25, 26]. Large sample investigations of the actual immunization state of psychiatric patients and its influential factors are still lacking. We conducted a survey in one of the top four national clinical research center of mental disorders in China during the COVID-19 pandemic. This study aimed to investigate the intention, vaccination status, and potential influencing factors of vaccination; evaluate the association between vaccination and psychological status among psychiatric patients [27]; find the imperfections in current vaccination programmes; and give them more reliable vaccination recommendations.

## Methods

### Participants

**Patients will be eligible to participate if they meet all of the following criteria**:

1. aged 12 years or older.
2. previously diagnosed with bipolar disorders, depressive disorders, anxiety disorders, obsessive-compulsive disorders, sleep disorders, schizophrenia, and other mental disorders, based on the *Diagnostic and Statistical Manual of Mental Disorders* (5th ed.; DSM-5; American Psychiatric Association, 2013).
3. able to read and understand these questions.
4. willing to participate in the survey.

**Caregivers will be eligible to participate if they meet all of the following criteria**:

1. aged ≥ 12.
2. immediate family members of psychiatric patients and should have lived with them for long periods.
3. capable of understanding and answering all the questions.
4. willing to participate in the survey.

**Patients and caregivers will be excluded if they had difficulty to concentrate on and complete the survey during the investigation.**

## Study design

The cross-sectional anonymous survey was designed for investigating the attitude of psychiatric patients and their family members towards COVID-19 vaccination and assessing the present psychiatric status of the participants. The face-to-face investigations recruited 1533 patients and 1034 family members in total between August 9 and August 24, 2021 at the psychiatric outpatient and inpatient department of the Second Xiangya Hospital of Central South University, China. During the post-peak period, the Chinese government expanded the population prioritized to COVID-19 vaccination, including frontline occupational stuff and teenagers aged between 12 and 17 [28, 29]. Informed consents were on the first page of the survey and included the aims, background and privacy protection of the study. The investigation would be started only after all the participants and the guardians of juveniles checked the agreement boxes on the informed consents. The study was approved by the Ethics Review Committee of Second Xiangya Hospital of Central South University (Reference number: (2021) National Ethic review [Section] No.(037). All the participants were required to finish the survey by themselves and they could consult with 4 researchers if they had question about the survey.

Two different versions of questionnaires were presented to psychiatric patients and their family members. Both comprised four parts: (1) basic information, including gender, age, job, marital status, economic status of their family, education level, residential place, diagnosis information of mental illness and other chronic diseases, present medical treatment (only for psychiatric patients); (2) perception of the COVID-19 vaccination, the infection status of people around, perceived possibilities of infection, channels of understanding information about vaccines; (3) attitudes towards vaccines, covering reasons for support and worry, potential influential factors of willingness, and the time of first dose of vaccination; and (4) present psychological status, including the 9-item depression module from the Patient Health Questionnaire (PHQ-9) and the Generalized Anxiety Disorder-7 items (GAD-7). The PHQ-9 is based directly on the nine diagnostic criteria for Major Depressive Disorder (MDD) in the DSM-IV, scoring each of the criteria as “0” (not at all) to “3” (nearly every day) during the last two weeks to represent mild, moderate, moderately severe and severe depression symptoms with total scores of 5, 10, 15, and 20 respectively [30]. PHQ-9 is also a validated screening tool with a cutoff of 10 or higher, sensitivity of 89.5% and specificity of 77.5% for the diagnosis of MDD for adolescents [31, 32]. The GAD-7 is a valid and efficient method for screening GAD and evaluating severity. Each of the seven items was scored from 0 to 3, so the GAD-7 scale score ranged from 0 to 21. The assessment criteria were “no anxiety” (score 0–4), “mild anxiety” (5–9), “moderate anxiety” (10–14), and “severe anxiety” (≥ 15) [33]. GAD-7 with a cutoff greater than 10 has a sensitivity of 97% and a specificity of 100% for diagnosing at least moderate anxiety in adolescents [34].

### Statistical analysis

SPSS 26.0 (IBM Corp) was used for data analysis. The significance level was set at P = 0.05. The original scores of the two measurement tools were normally distributed; therefore, these data were presented as mean with standard deviation (SD). The vaccination population was presented as numbers and percentages. The Fisher exact tests were applied to determine the statistical significance of the vaccination status at different variable levels, covering demographic characteristics and related influential factors as univariate analysis. To determine the potential risk factors for vaccination status, binary logistic regression analysis was performed as multivariate analysis. The regression models included age, gender, marital status, fertility, residence, educational level, occupation, psychiatric diagnosis, patient type, preferred vaccines and intention of vaccination as independent variables and vaccination status as dependent variables. The forward selection method was then applied to incorporate all significant variables. The associations between risk factors and outcomes were presented as odds ratios (ORs) with 95% confidence intervals, adjusting for age.

## Results

### Demographic characteristics

Overall, among the 2576 recruited participants, 1328 psychiatric patients and 922 family members completed the survey. The response rates for patients and family members were 86.6% and 89.2%, respectively. Of the 1328 patients with a mean age of 27.65 (± 12.26), 780 (58.7%) were aged 18–34 years, 876 (66.0%) were female, 885 (66.6%) were unmarried, 929 (69.9%) were childless, 546 (41.1%) were living in the city, 639 (48.1%) had a bachelor degree level of education or above, 604 (45.5%) had a job, and 392 (29.5%) had a salary between 741.93-1483.86 dollars per month. The primary psychiatric diagnoses of patients were depression disorder (30.9%), bipolar disorder (22.4%), schizophrenia (13.3%), and anxiety disorder (11.6%). 70% of the patients were suffering from one psychiatric condition, while 30% patients comorbid more than one psychiatric disorder. A total of 198 psychiatric patients had comorbid somatic diseases, including cardiovascular disease (4.1%), endocrine disease (2.3%), and other physical diseases. Most patients were outpatients (79.8%) and were willing to be vaccinated (85.5%). While 38.6% of patients preferred inactivated vaccines, 39.1% preferred not to choose which kind of vaccine. The basic characteristics of family members were shown in Supplementary Table 1.

As for the current mood status, 61.9% (822/1328) patients scored over the cutoff point on PHQ-9 with a standardized score of 10.42 ± 8.18, comprising mild depression (17.2%), moderate depression (14.9%), severe depression (29.8%). The GAD-7 was used to assess anxiety levels and showed that 51.9% (689/1328) of patients with a standard score of 7.72 ± 6.61 could be considered to have anxiety, with 33% (440/1328) reporting moderate to severe anxiety. (Table 1)

**Table 1:** Basic characteristics and vaccination rates of patients

	Patients	Vaccinated patients	Vaccination rate	P-value
	(N = 1328)	(N = 921)		
	n/N (%)	n/N (%)		
Age, mean (SD)	27.65(12.26)	26.76(11.13)	NA^a^	
Age group				< 0.001
12-17y	230(17.3%)	147(16.0%)	147/230(63.9%)	
18-34y	780(58.7%)	582(63.2%)	582/780(74.6%)	
35-49y	228(17.2%)	147(16.0%)	147/228(64.5%)	
50-64y	76(5.7%)	41(4.4%)	41/76(53.9%)	
≥ 65y	14(1.1%)	4(0.4%)	4/14(28.6%)	
Gender				0.006
Male	452(34.0%)	291(31.6%)	291/452(64.4%)	
Female	876(66.0%)	630(68.4%)	630/876(71.9%)	
Marital status				0.009
Unmarried	885(66.6%)	630(68.4%)	630/885(71.2%)	
Married	394(29.7%)	263(28.6%)	263/394(66.7%)	
Divorce	47(3.5%)	27(2.9%)	27/47(57.4%)	
Unfilled	2(0.2%)	1(0.1%)	1/2(50.0%)	
Fertility				0.019
Already birth	397(29.9%)	257(27.9%)	257/397(64.7%)	
Not yet birth	929(69.9%)	662(71.9%)	662/929(71.3%)	
Unfilled	2(0.2%)	2(0.2%)	2/2(100.0%)	
Urban and rural resource				< 0.001
City	546(41.1%)	409(44.4%)	409/546(74.9%)	
Town	322(24.2%)	229(24.8%)	229/322(71.1%)	
Countryside	457(34.4%)	281(30.5%)	281/457(61.5%)	
Unfilled	3(0.2%)	3(0.3%)	2/3(66.7%)	
Education level				< 0.001
Primary school and below	36(2.7%)	18(1.9%)	18/36(50.0%)	
junior middle school	240(18.1%)	137(14.9%)	137/240(57.1%)	
College or vocational school	405(30.5%)	264(28.7%)	264/405(65.2%)	
Bachelor degree or above	639(48.1%)	497(54.0%)	497/639(77.8%)	
Unfilled	8(0.6%)	5(0.5%)	5/8(62.5%)	
Occupation				0.001
Employed	604(45.5%)	388(42.1%)	388/604(64.2%)	
Unemployed	262(19.7%)	189(20.5%)	189/262(72.1%)	
Student	460(34.6%)	342(37.1%)	342/460(74.3%)	
Unfilled	2(0.2%)	2(0.2%)	2/2(100.0%)	
Family income				0.110
Less than $296.77 per month	177(13.3%)	110(11.9%)	110/177(62.1%)	
$ 296.77-741.93 per month	38(2.9%)	25(2.7%)	25/38(65.8%)	
$ 741.93-1483.86 per month	392(29.5%)	280(30.4%)	280/392(71.4%)	
$ 1483.86-2967.71 per month	258(19.4%)	182(19.8%)	182/258(70.5%)	
More than $ 2967.71 per month	162(12.2%)	121(13.1%)	121/162(74.7%)	
Unfilled	301(22.7%)	203(22.1%)	203/301(67.4%)	
Psychiatric diagnosis				0.022
Bipolar disorder	297(22.4%)	220(23.9%)	220/297(74.1%)	
Depression disorder	410(30.9%)	303(32.9%)	303/410(73.9%)	
Anxiety disorder	154(11.6%)	102(11.1%)	102/154(66.2%)	
Obsessive-compulsory disorder	33(2.5%)	28(3.0%)	28/33(84.8%)	
Sleep disorder	69(5.2%)	55(6.0%)	55/69(79.7%)	
Schizophrenia	177(13.3%)	91(9.9%)	91/177(51.4%)	
Other psychiatric disorders	188(14.2%)	122(13.2%)	122/188(64.9%)	
Numbers of psychiatric disease				
One	929(70.0%)	651(70.7%)	651/929(70.1%)	0.072
Two	267(20.1%)	190(20.6%)	190/267 (71.2%)	
Three and above	132(9.9%)	80(86.7%)	80/132(60.6%)	
Somatic disease				0.470
Cardiovascular disease	54(4.1%)	32(3.5%)	32/54(59.3%)	
Endocrine disease	31(2.3%)	15(1.6%)	15/31(48.4%)	
Respiratory disease	11(0.8%)	6(0.6%)	6/11(54.5%)	
Tumor	8(0.6%)	3(0.3%)	3/8(37.5%)	
Others	94(7.0%)	59(6.4%)	59/94(62.8%)	
None	1130(85%)	806(87.5%)	806/1130(71.3%)	
Patient type				< 0.001
inpatient	222(16.7%)	117(12.7%)	117/222(52.7%)	
outpatient	1060(79.8%)	786(85.3%)	786/1060(74.2%)	
unfilled	46(3.5%)	18(2.0%)	18/46(39.1%)	
Intention of vaccination				< 0.001
willing	1135(85.5%)	880(95.5%)	880/1135(77.5%)	
unwilling	48(3.6%)	10(1.1%)	10/48(20.8%)	
Not sure	64(4.8%)	8(0.9%)	8/64(12.5%)	
Indifferent	44(3.3%)	19(2.1%)	19/44(43.2%)	
Unfilled	37(2.8%)	4(0.4%)	4/37(10.8%)	
Preferred vaccine				< 0.001
Adenovirus-vectored vaccine (one dose)	123(9.3%)	77(8.4%)	77/123(62.6%)	
Inactivated vaccine (two doses)	512(38.6%)	417(45.3%)	417/512(81.4%)	
Recombinant protein vaccine (three doses)	173(13.0%)	135(14.6%)	135/173(78.0%)	
Unknow	520(39.1%)	292(31.7%)	292/520(56.2%)	
PHQ-9^b^				0.582
No depression	384(28.9%)	269(29.2%)	269/384(70.1%)	
Mild depression	228(17.2%)	168(18.2%)	168/228(73.7%)	
Moderate depression	198(14.9%)	148(16.1%)	148/198(74.7%)	
Severe depression	396(29.8%)	291(31.6%)	291/1328(73.5%)	
Unfilled	122(9.2%)	45(4.9%)	45/122(36.9%)	
Mean (SD)	10.42(8.18)	10.56(8.16)	NA^a^	
GAD-7^c^				0.115
No anxiety	469(35.3%)	321(34.9%)	321/469(68.4%)	
Mild anxiety	249(18.8%)	189(20.5%)	189/249(75.9%)	
Moderate anxiety	207(15.6%)	156(16.9%)	156/207(75.4%)	
Severe anxiety	233(17.5%)	169(18.3%)	169/233(75.8%)	
Unfilled	170(12.8%)	86(9.3%)	86/170(50.1%)	
Mean (SD)	7.72(6.61)	7.99(6.59)	NA^a^	
Total	NA^a^	NA^a^	921/1328(69.4%)	

^a^: Not Applicable

^b^: Patient Health Questionnaire-9 items

^c^: Generalized Anxiety Disorder-7 items

### Vaccination status and vaccination rates

Among the patients, 69.4% (921/1328) had been vaccinated at the time of participating in the survey, which was low when compared to their family members (89.8%, Supplementary Table 1). The vaccination rate of patients between 18 and 34 years old was 582/780 (74.6%), the highest among all age groups, higher than that of adolescents (63.9%). When ≥ 35 years, the upper age group had lower vaccination rates. There are fewer vaccinated patients living in the countryside (61.5%) than in the city (74.9%) and in town (71.1%). The vaccination rate of patients with a bachelor’s degree or above was 77.8%, followed by college or vocational school graduates (65.2%), junior middle school students (57.1%), and primary school students and below (50.0%). The unemployed had a higher vaccination rate (72.1%) than the employed (64.2%). After taking the primary psychiatric diagnosis into consideration, 51.4% of patients with schizophrenia were vaccinated, which was considerably lower than those with other psychiatric disorders. 71.3% patients with no somatic disease accepted vaccination, which was higher than those with endocrine disease (48.4%), respiratory disease (54.5%), and cardiovascular disease (59.3%). The vaccination rate in hospitalized patients (52.7%) was much lower than outpatients (74.2%). 77.5% patients who were willing to be vaccinated had already been administered the vaccine, while 20.8% of patients were unwilling to accept inoculation. Patients who preferred inactivated vaccines had the highest vaccination rates (81.4%), followed by those who preferred recombinant protein vaccines (78.0%).

Univariate analysis showed that patients who were 18–50, female, unmarried, not yet birth, unemployed, outpatients, willing to get vaccinated, lived in the city, had an educational level of bachelor’s degree or above, preferred inactivated vaccine (two doses), and those with psychiatric disorders except for schizophrenia and with no physical diseases, were more likely to be vaccinated (p < 0.05; Table 1).

### Factors associated with vaccination status

The multivariate binary logistic regression analysis (Table 2) found that after controlling for confounders, only finishing junior middle school was associated with lower vaccination rates (OR = 0.58; 95% CI = 0.37–0.93; P < 0.05) compared to graduating from universities. Schizophrenia was significantly associated with not being inoculated (OR = 0.38; 95% CI = 0.19–0.75; P < 0.05) when compared to other primary psychiatric diseases. Hospitalization was associated with being unvaccinated (OR = 0.41; 95% CI = 0.27–0.60; P < 0.05). The attitudes toward vaccines were obviously associated with vaccination status as patients who were willing to be vaccinated were more likely to get inoculated (OR = 5.33; 95% CI = 2.59–10.96; P < 0.05) and patients who were unwilling (OR = 0.24; 95% CI = 0.08–0.71; P < 0.05) and uncertain (OR = 0.14; 95% CI = 0.05–0.42; P < 0.05) about vaccination were less likely to be inoculated compared to those who were indifferent about vaccines. Inactivated vaccine (two doses) (OR = 1.58; 95% CI = 1.10–2.29; P < 0.05) and recombinant protein vaccine (three doses) (OR = 1.89; 95% CI = 1.13–3.16; P < 0.05) were associated with receiving the vaccination project.

**Table 2:** Binary logistic regression analysis of personal variables associated with vaccination status

Variables and variable levels	Association with vaccination	
	aOR^a^ (95% CI)	P-value
Education level		
Primary school and below	0.82(0.28–2.38)	0.715
Junior middle school	0.58(0.37–0.93) *	0.023
College or vocational school	0.80(0.54–1.20)	0.280
Bachelor degree or above	Reference	
Psychiatric diagnosis		
Bipolar disorder	0.99(0.51–1.93)	0.982
Depression disorder	1.04(0.55–1.97)	0.915
Anxiety disorder	0.76(0.37–1.57)	0.759
Obsessive-compulsory disorder	2.91(0.77–11.02)	0.115
Sleep disorder	1.26(0.50–3.17)	0.631
Schizophrenia	0.38(0.19–0.75) **	0.005
Other psychiatric disorders	Reference	
Patient type		
Inpatient	0.41(0.27–0.60) **	< 0.001
Outpatient	Reference	
Intention of vaccination		
Willing	5.33(2.59–10.96) **	< 0.001
Unwilling	0.24(0.08–0.71) *	0.010
Not sure	0.14(0.05–0.42) **	< 0.001
Indifferent	Reference	
Vaccine type (dosage form)		
Adenovirus-vectored vaccine (one dose)	0.74(0.45–1.23)	0.250
Inactivated vaccine (two dose)	1.58(1.10–2.29) *	0.015
Recombinant protein vaccine (three dose)	1.89(1.13–3.16) *	0.015
Unknow	Reference	

^a^: Adjusted for age

* : P < 0.05

** : P < 0.0

Another binary logistic regression analysis (Table 3) showed a significant association between vaccination time and psychiatric status after adjusting for gender, marital status. Compared to patients vaccinated over a month ago, patients vaccinated in the recent month were less likely to have depression (OR = 0.63; 95% CI = 0.41–0.98; P < 0.05) and anxiety (OR = 0.40; 95% CI = 0.25–0.63; P < 0.05).

**Table 3:** The association between vaccination time and mood status

	No(%) with depression	aOR^a^(95%CI)	P	No(%) with anxiety	aOR^a^(95%CI)	P
Vaccination time						
In the recent month	177/222(79.7%)	0.63(0.41–0.98)*	0.040	174/222(78.4%)	0.40(0.25–0.63)**	< 0.001
More than one month	519/629(82.5%)	reference		542/629(86.2%)	reference	
Psychotropic drug use						
No	189/266(71.1%)	1.74(1.00-3.03)	0.052	168/266(63.2%)	1.72(0.95–3.13)	0.074
Yes	602/983(61.2%)	reference		518/983(52.7%)	reference	

^a^: Adjusted for gender, marital status

* : P < 0.05

** : P < 0.01

The results of the univariate and multivariate analysis above showed that junior middle school degree, schizophrenia, hospitalization, the attitudes towards vaccination and preferred vaccines were independent influential factors of vaccination status.

### Reasons and factors for vaccination attitudes

Table 4 lists the supports and worries of patients and their family members for vaccination. The top three reasons for unwillingness in the patients were “concerns about effectiveness and safety” (26.8%), “concerns about exacerbating psychiatric disorders for self” (17.6%), and “concerns about exacerbating side effects of psychotropic drugs” (7.9%). Only a small number of patients were frustrated due to their psychiatric symptoms: “they don’t want to live at all” (8.0%), “concerning that vaccines are useless and infection is absolutely” (5.5%), “they have no interest in everything including vaccines” (4.8%). Family members, worried more about “effectiveness and safety” (34.5%), “long waiting time after appointment” (5.3%), “media and vaccinated people declare negative feedback” (4.6%). With regard to promoting vaccination intention, “preventing COVID-19 infection effectively” (76.9%), “reducing the risk of infection of surrounding people” (62.9%), and “providing convenience for commuting and travelling” (61.5%), ranked high among patients, as well as family members, the corresponding proportions of which were 80.9%, 66.0% and 60.4% respectively.

**Table 4:** Supports and worries about vaccination of the participants

Reasons of willingness and unwillingness	Patients (n = 1328) n(%)	Family members (n = 922) n(%)
Supports		
Prevent COVID-19 infection effectively	1071(76.6%)	746(80.9%)
Decrease the risk of infection of surrounding people	817(61.5%)	557(60.4%)
Provide convenience for commuting and travelling	835(62.9%)	609(66.0%)
Improve mood state	336(25.3%)	NA^1^
Take care of patients more easily and conveniently.	NA	162(17.6%)
Worries		
Concerns about effectiveness and safety	356(26.8%)	318(34.5%)
Concerns about exacerbating psychiatric disorders for self	234(17.6%)	NA
Concerns about exacerbating adverse effects of psychotropic drugs	105(7.9%)	NA
Media and vaccinated people declare negative feedback	35(2.6%)	42(4.6%)
Long waiting time after appointment	66(5.0%)	49(5.3%)
No necessity for vaccination since pandemic controlled	21(1.6%)	3.7(0.4%)
No interest in everything including vaccines	64(4.8%)	NA
Concern that vaccines could result in disability or death	28(2.1%)	NA
Concern that vaccines are useless and infection is absolutely	73(5.5%)	NA
Don’t want to live at all	106(8.0%)	NA
Some voice in mind when alone say cannot get vaccinated	16(1.2%)	NA
Vaccines could be a tool for stalking	9(0.7%)	NA

^1^: Not Applicable

## Discussion

To our knowledge, this is the first large sample study of vaccination status and its potential factors in people with psychiatric disorders and their family members in China. Our findings showed that most psychiatric patients were willing to be inoculated with COVID-19 vaccines despite not having been vaccinated, especially those have been hospitalized or those with a severe mental illness. The results could reveal the unsatisfied demands for vaccination in psychiatric patients compared with the general population and provide governments worldwide with evidence to increase vaccination rates in psychiatric patients.

This cross-sectional study showed that 85.5% of patients were willing to be vaccinated, lower than their family members (93%, Supplementary Table 1) and the general population in China (91.3%) [35].This indicated a high COVID-19 vaccination willingness in people with mental illness and is consistent with previous reports in both China and other countries. A preliminary online survey conducted in January showed that 77.8% of psychiatric patients intended to receive COVID-19 vaccination [26]. Another study also revealed a high acceptance rate of 96.2% and willingness to pay for vaccines in patients with depression and anxiety disorders in China [25]. Similar results of attitudes towards vaccination in people with mental disorders were also confirmed in countries such as Denmark (84.8%) and Belgium (93%) [36]. Although these studies were conducted during different periods of the coronavirus pandemic, the need for COVID-19 vaccine uptake in psychiatric patients is undoubtedly great. However, we investigated the vaccination status of psychiatric patients and found that only 69.4% of patients were already vaccinated, which was much lower than their family members (89.8%) and the general population (78.0%) in China, reminding us that the demand for vaccination in some psychiatric patients was ignored.

To reveal the reasons for lower vaccination willingness and lower vaccination rates in patients with mental illness than in the general population, we classified their concerns and found that, in addition to some common primary causes such as uncertainty about effectiveness and safety, long waiting periods, and passive feedback on vaccines, patients were mainly confused by whether the possible side effects of COVID-19 vaccines would lead to exacerbation of psychiatric disorders and adverse drug reactions of psychotropics. In addition, 25% of patients showed a promotion of willingness if vaccines could improve their mood status. Besides, a small part of worries about vaccines resulted from mental health symptoms of patients, including suicidal thoughts, negative cognition, decreased interest, delusion, and hallucination. Existing studies also indicated that alcohol, tobacco and substance use disorders were associated with vaccine hesitancy in patients with mental illness, especially in social isolation due to increased barriers to receive medical care [37–39]. Besides, perceived social isolation accompanied with changes in daily life, loneliness, unemployment, economic hardship and the pain of losing family members would result in clinically significant mental health problems like distress and depression by promoting tonic sympathetic tonus and HPA activation as well as lowering inflammatory regulation, immune response, and expression of glucocorticoid responses related genes [40–42]. Mental health conditions including depression, anxiety, and fear for infection were associated with unwillingness to receive vaccination against COVID-19 [43].

The vaccination rate of people with mental illness is associated with several personal variables. Patients who had junior middle school education or below, who had schizophrenia, were inpatients and were reluctant or unsure of vaccination were less likely to receive vaccines against coronavirus. Patients with schizophrenia had higher hospitalization (45.7%) and lower vaccination rates (51.4%, OR = 0.38; 95% CI = 0.19–0.75; P < 0.05) than those with other psychiatric diseases. Previous studies and experiences have shown that the barriers to accessing COVID-19 vaccine for severe psychiatric patients comprised individual-level barriers (their incorrect estimation of contracting risk for self, poor social support, and negative perception about coronavirus), and systemic barriers (vaccine education, policies, monitoring programs, structural resources, and cost) [44]. A study in Israel showed that vaccines had sufficient protective effects against COVID-19 in patients with schizophrenia. Thus, future national vaccination plans should aim to actively reach out to people with schizophrenia to solve the inequitable allocation of COVID-19 vaccines [45]. Hospitalized patients had lower vaccine uptake rates (52.7%; OR = 0.41; 95% CI = 0.27–0.60; P < 0.05) than outpatient patients (74.2%). Inpatients are more vulnerable to COVID-19 since they usually live in closed environments, leading to a cluster of in-hospital infections if someone contracted COVID-19 before admission to the hospital [19, 46]. Therefore, we propose that patients should also be included in the priority vaccination group and that targeted immunization programs should be carried out. Patients with lower educational levels had lower vaccination rates, probably owing to their misunderstanding about COVID-19 and misconceptions about vaccines. Patients who preferred inactivated vaccines (two doses) and recombinant protein vaccines (three doses) had a higher tendency to receive vaccination, possibly because during the early vaccination project, vaccine selection were mainly based on the local repertories and we might speculate from the vaccination policy that inactivated vaccines (two doses) and recombinant protein vaccines (three doses) was on the market for a longer time and people had more confidence in them due to the promotion of the government in China. After controlling confounders, we found that patients who had been vaccinated in a recent month had lower tendency to get depression (OR = 0.63; 95% CI = 0.41–0.98; P < 0.05) and anxiety (OR = 0.40; 95% CI = 0.25–0.63; P < 0.05). An online study in Polish showed that fully vaccinated people presented lower levels of anxiety than those who were partly vaccinated or not vaccinated [47]. A cross-sectional survey in China also implied that COVID-19 vaccination could relieve anxiety and depression in vaccinated individuals [48]. A cohort study in American revealed that COVID-19 vaccination could ameliorate distress and decline perceived risk of infection, hospitalization and death [49]. Since the COVID-19 pandemic was associated with a higher prevalence of psychiatric disorder symptoms both in psychiatric patients and the general population, vaccine uptaking might alleviate their psychological burden and improve their mental health conditions temporarily. Except for the short-term direct effects of significant improvements in mental health on people already vaccinated, there are also large contributions to unvaccinated people who might benefit from reduced worries about infection and increased public beliefs in vaccines, which could encourage them to get vaccinated, facilitate herd immunization, and stimulate economic recovery [50]. Moreover, social isolation might influence the demand for vaccination as well.

Patients with severe mental illness were reported to have a higher risk of COVID-19 infection and mortality since they were likely to live in crowded and unsafe environments because of poor financial condition, unstable symptoms and unwilling to seek medical aid for fear of stigma and discrimination [51, 52]. With the advocacy of prioritizing COVID-19 vaccination for psychiatric patients, some countries have rolled out vaccination drives for outpatients with severe mental illness, including Denmark, Germany, the Netherland and the UK [44, 53]. Our research could provide some evidence for maximizing immunization programs. For governments and healthcare commission, policies are needed for the vaccination assurance of patients with severe psychosis and patients living in crowded wards who are isolated from their families. Mental health professionals should either advise every patient to receive vaccination on time or raise awareness about COVID-19 among their patients and caregivers, especially those dealing with schizophrenia, and poor education. Family members should persuade patients to receive COVID-19 vaccines and relieve their stress before and after vaccination. Nevertheless, some situations that may contribute to adverse reactions after COVID-19 vaccination should not be ignored. Since up to 1% of people might experience allergic symptoms, including itchy skin, rash, or hives, patients with unstable psychiatric conditions should be cautiously evaluated before vaccination. Use of sensitizing drugs such as lamotrigine for a long time should be carefully monitored in cases of severe anaphylaxis to avoid drug eruption, angioedema, and inflammation of the nervous system [23].

This study had several limitations. First, the research sample was not fully presentative resulting in selection bias. The sample was better educated because patients with poor education couldn’t finish the questionnaire by themselves and understand the meaning of our investigation since we did not provide any intervention or examination, leading to their refusal to participate in the study. The survey was also a single center study and could not represent the national situation. In addition, volunteer bias of higher vaccination willingness and vaccination rates may be present. Second, the cross-sectional study could not show the changes in psychological status in psychiatric patients before and after vaccination. Future self-controlled studies should be conducted to confirm the emotional changes associated with vaccination in people with mental disorders.

## Conclusion

In this study of psychiatric patients receiving COVID-19 vaccines in China, high acceptance and relative low vaccine uptake rates was reported. Besides conventional public health measures, imperative targeted vaccination strategies on people with mental disorders should be practiced. Furture vaccination schedule should ensure effective access to vaccines in patients with schizophrenia, hospitalized and poor education.

## Electronic supplementary material

Below is the link to the electronic supplementary material.

Supplementary Material 1: Basic characteristics and vaccination rates of family members

## Data Availability

The datasets used and analyzed during the current study are available from the corresponding authors on reasonable request.

## Abbreviations

COVID-19: Coronavirus Disease 2019.
PHQ-9: Patient Health Questionnaire-9.
GAD-7: Generalized Anxiety Disorder-7 items.
WHO: World Health Organization.
NHCC: National Health Commission of China.
DSM: Diagnostic and Statistical Manual of Mental Disorders.
PTSD: post-traumatic stress disorder.
MDD: Major Depressive Disorder.
OCD: obsessive-compulsive disorder.
