# Supplementary material for: COVID-19 vaccination coverage among patients with psychiatric disorders in China during the pandemic: a cross-sectional study
Source: BMC Psychiatry. 2022 Oct 26;22:659. doi: 10.1186/s12888-022-04271-w (PMC9597954; doi:10.1186/s12888-022-04271-w)
Supplement: Supplementary file 1 — Supplementary Material 1: Basic characteristics and vaccination rates of family members [file 12888_2022_4271_MOESM1_ESM.doc]

**Supplementary Table 1.** **Basic characteristics and vaccination rates of family members**.

|  | Family members | Vaccinated family members (vaccination rate) |
| --- | --- | --- |
|  | (N=922) | (N=828) |
|  | n/N (%) | n/N (%) |
| Age, mean (SD) | 38.59(11.936) | 38.68(11.589) |
| PHQ-9^b^, mean (SD) | 2.44(4.036) | 2.35(3.960) |
| GAD-7^c^, mean (SD) | 1.96(3.222) | 1.92(3.183) |
| Gender |  |  |
| Male | 376(40.8%) | 336/376(89.4%) |
| Female | 544(59.0%) | 491/544(90.3%) |
| Unfilled | 1(0.2%) | 1/1(100%) |
| Marital status |  |  |
| Unmarried | 193(20.9%) | 169/193(87.6%) |
| Married | 691(74.9%) | 627/691(90.7%) |
| Divorce | 29(3.1%) | 26/29(89.7%) |
| Unfilled | 9(0.9%) | 6/9(66.7%) |
| Fertility |  |  |
| Already birth | 691(74.9%) | 629/691(91.0%) |
| Not yet birth | 227(24.6%) | 198/227(87.2%) |
| Unfilled | 4(0.4%) | 1/4(25%%) |
| Urban and rural resource |  |  |
| City | 395(42.8%) | 346/395(87.6%) |
| Town | 193(20.9%) | 185/193(95.9%) |
| Countryside | 328(35.6%) | 300/328(91.5%) |
| Unfilled | 6(0.7%) | 6/6(100%) |
| Education level |  |  |
| Primary school and below | 44(4.8%) | 43/44(97.7%) |
| junior middle school | 159(17.2%) | 144/159(90.6%) |
| College or vocational school | 213(23.1%) | 181/213(85.0%) |
| Bachelor degree or above | 499(54.1%) | 456/499(91.4%) |
| Unfilled | 7(0.8%) | 4/7(57.1%) |
| Occupation |  |  |
| Employed | 667(72.3%) | 613/667(91.9%) |
| Unemployed | 188(20.4%) | 160/188(85.1%) |
| Student | 62(6.7%) | 52/62(83.9%) |
| Unfilled | 5(5.4%) | 3/5(60%) |
| Family income |  |  |
| Less than $ 296.77 per month | 88(9.5%) | 81/88(92.0%) |
| $ 296.77-741.93per month | 219(23.8%) | 200/219(91.3%) |
| $ 741.93-1483.86 per month | 261(28.3%) | 238/261(91.2%) |
| $ 1483.86-2967.71 per month | 196(21.3%) | 179/196(91.3%) |
| More than $ 2967.71 per month | 109(11.8%) | 96/109(88.1%) |
| Unfilled | 49(5.3%) | 34/49(69.4%) |
| Intention of vaccination |  |  |
| willing | 857(93.0%) | 810/857(94.5%) |
| unwilling | 14(1.5%) | 5/14(35.7%) |
| Not sure | 10(1.1%) | 4/10(40.0%) |
| Indifferent | 7(0.8%) | 3/7(42.9%) |
| Unfilled | 34(3.7%) | 6/34(17.6%) |
| Preferred vaccine |  |  |
| Adenovirus-vectored vaccine (one dose) | 72(7.8%) | 67/72(93.1%) |
| Inactivated vaccine (two doses) | 516(56.0%) | 508/516(98.4%) |
| Recombinant protein vaccine (three doses) | 146(15.8%) | 140/146(95.9%) |
| Unknow | 188(20.4%) | 113/188(60.1%) |
| Total | NA^a^ | 828/922(89.8%) |

^a^：Not Applicable

^b^：Patient Health Questionnaire-9 items

^c^：Generalized Anxiety Disorder-7 items
